# Targeting the gut-liver axis in cholangiocarcinoma: mechanisms, therapeutic advances, and future directions

**DOI:** 10.3389/fonc.2025.1646897

**Published:** 2025-09-12

**Authors:** Lu Wang, Weiwei Qiao, Xiaowen Zhen, Yeqiong Zhang, Zhiwei Dong

**Affiliations:** ^1^ Department of Diagnostics, Second School of Clinical Medicine, Binzhou Medical University, Yantai, Shandong, China; ^2^ Department of Infectious Diseases, Third Affiliated Hospital of Sun Yat-sen University, Guangzhou, Guangdong, China; ^3^ Department of Surgery, Faculty of Medicine, The Chinese University of Hong Kong, Hong Kong, Hong Kong SAR, China

**Keywords:** gut-liver axis, cholangiocarcinoma, gut microbiota, dysbiosis, bile salt, therapy, clinical translation

## Abstract

Cholangiocarcinoma (CCA), a highly aggressive biliary tract malignancy, exhibits rising incidence rates and an extremely poor prognosis. Recent studies reveal that gut-liver axis dysregulation drives CCA progression through gut microbiota dysbiosis, bile acid (BA) metabolic disturbances, and immune microenvironment remodeling. Clinical evidence highlights significant alterations in the gut and biliary microbial composition of CCA patients, which correlate with tumor stage, vascular invasion, and survival outcomes. Dysregulated BA metabolism in CCA, characterized by accumulation of primary conjugated BAs, promotes tumor invasiveness via interaction with specific BA receptors and fosters an immunosuppressive microenvironment. Emerging therapeutic strategies include antibiotics for pathogenic microbiota modulation, probiotics for microbial homeostasis restoration, fecal microbiota transplantation, and BA pathway modulators. Future directions necessitate integrating synthetic biology (engineered microbiota), multi-omics, and artificial intelligence to develop precision therapies. Targeting the gut-liver axis offers novel therapeutic perspectives for CCA; however, clinical translation demands deeper mechanistic insights and standardized protocols to address challenges such as microbiota heterogeneity and receptor signaling duality.

## 1 Introduction

Cholangiocarcinoma (CCA), an aggressive malignant tumor of the bile ducts, exhibits distinct epidemiological trends across subtypes (1). CCA is anatomically classified as intrahepatic (iCCA) or extrahepatic (eCCA), with eCCA further subdivided into perihilar and distal subtypes based on their location relative to the cystic duct (2). Globally, CCA incidence is increasing, notably iCCA (3, 4), which is the second most common cause of primary liver cancer, after hepatocellular carcinoma (HCC) (5). The peak incidence of CCA ocures between 60 and 70 years, more commonly arising in males (6). While the risk factors for CCA subtypes are distinct, primary sclerosing cholangitis (PSC) is a well-established common risk factor for both iCCA and eCCA (7). The persistently poor prognosis (5-year survival <15%) largely stems from late-stage diagnosis, as the asymptomatic of disease onset often precludes timely therapeutic intervention (8). Current management of CCA remains clinically challenging. Surgical resection is the only curative option, which is limited to the majority patients (9). Patients with advanced unresectable or metastatic CCA face constrained therapeutic efficacy from systemic therapies, demonstrating median overall survival (OS) of 6-18 months (10, 11). Recent advancements incorporating chemoimmunotherapy regimens [e.g., gemcitabine/cisplatin combined with pembrolizumab (12)] and molecularly targeted agents [e.g., FGFR2 inhibitors synergized with cisplatin (13)] have broadened treatment paradigms. Nevertheless, clinical outcomes persist below expectations, with 12-month survival rates remaining under 40% in advanced-stage cohorts. Addressing the aggressive biology and molecular heterogeneity necessitates prioritized development of innovative neoadjuvant approaches leveraging multi-omics platforms to establish biomarker-directed therapeutic algorithms.

The gut-liver axis emerges as a master regulator of hepatobiliary disorders, governed by reciprocal signaling between hepatocytes, cholangiocytes, and intestinal microbiota (14, 15). This cellular triad drives disease pathogenesis through three interlinked mechanisms: microbiota-derived metabolite reprogramming, dysregulated bile acid (BA) enterohepatic cycling, and immune niche remodeling (16–18). Accumulating evidence establishes gut microbiota dysbiosis as a critical modulator of CCA pathogenesis and progression (19, 20). Pathobiont-derived metabolites translocate across the compromised intestinal barrier, activating hepatic Kupffer cells (KCs) to amplify pro-inflammatory cascades, thereby fueling hepatobiliary inflammation and fibrotic preconditioning (21). A critical component of this bidirectional interaction is the BA enterohepatic circulation. Bidirectional crosstalk emerges between microbial communities and BA metabolism, where altered BA composition and impaired excretion through BA specific receptor pathways perpetuate cholangitis-driven carcinogenesis (22). Concurrently, preclinical and clinical evidence converges to nominate the gut-liver axis as a high-value therapeutic frontier in CCA. This review will examine molecular mechanisms and clinical evidence linking the gut-liver axis to CCA progression. We will also evaluate dual targeting of gut microbiota and BA-associated signaling pathways as potential therapeutic strategies for CCA and propose the future research priorities and current translation challenges.

## 2 The gut-liver axis

**Figure 1:**
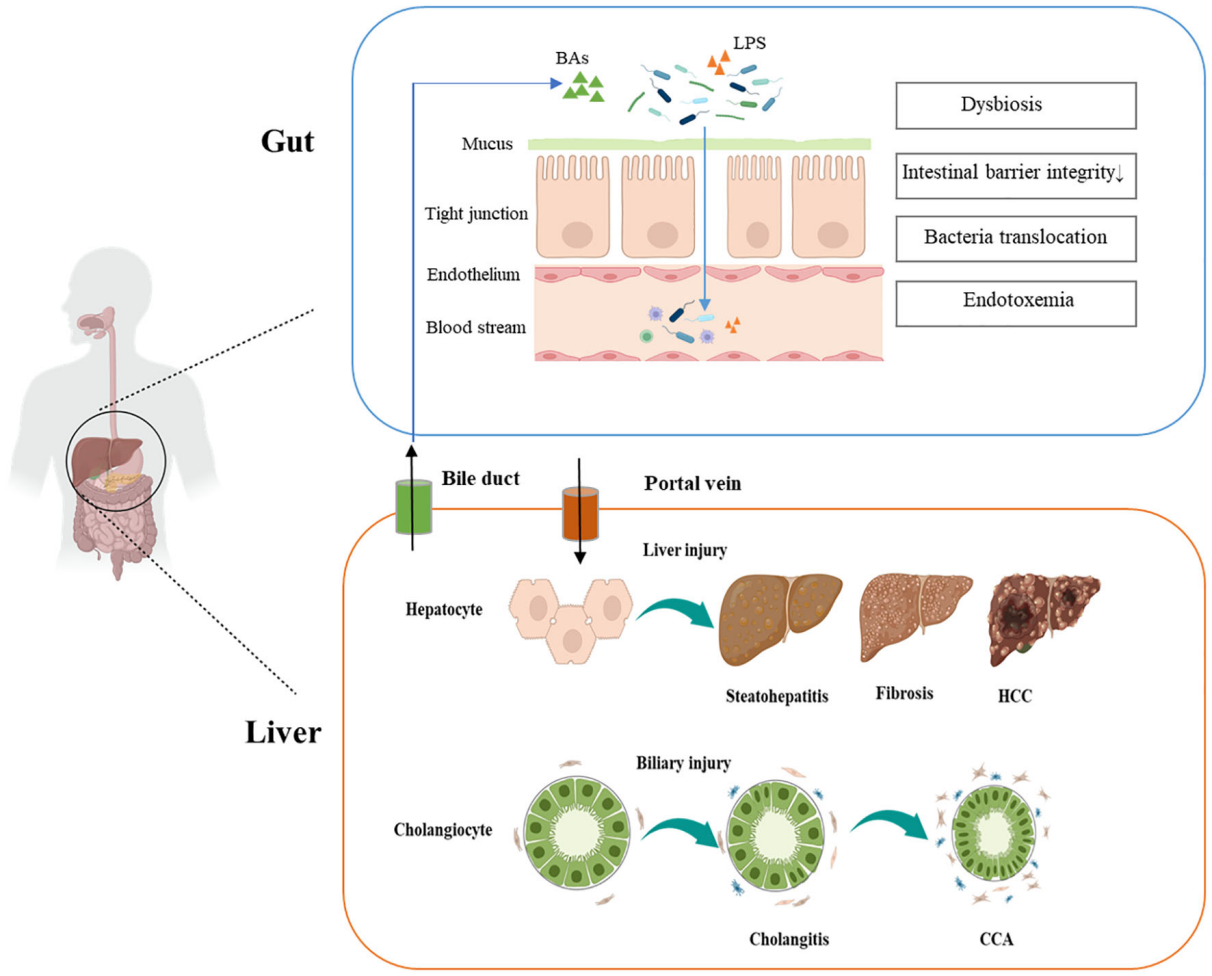
Gut–liver axis pathogenesis in cholangiocarcinoma and hepatocellular carcinoma.

The gut-liver axis orchestrates a bidirectional crosstalk network that coordinates environmental exposures, microbial dynamics, and host signaling to govern gastrointestinal homeostasis and disease progression (**Figure 1**). Mechanistically, disruption of this axis—characterized by intestinal barrier failure and microbial dysbiosis—promotes bacterial translocation into the biliary tract, where TLR activation and subsequent nuclear factor kappa-B(NF-κB) signaling drive immune-mediated cholangitis while impairing mucosal integrity (23). Notably, the gut epithelial tight junctions serve as a critical physical barrier against microbial invasion. Early perturbations of gut-liver axis homeostasis frequently originate from dietary insults or antibiotic-induced dysbiosis, which compromise tight junction integrity and initiate intestinal hyperpermeability (24). This breach facilitates systemic translocation of microbial metabolites—notably lipopolysaccharides (LPS)—from the portal circulation into hepatic tissues, triggering hepatic KCs to amplify pro-inflammatory cytokine production via coordinated activation of Toll-like receptor 4 (TLR4)/MyD88 signaling and IL-6/STAT3 signaling cascades. Such chronic inflammatory signaling drives both progressive hepatobiliary injury and direct oncogenic transformation through two interconnected mechanisms: (1) initiating oxidative DNA damage-induced genomic instability; and (2) activating dysregulated proliferative signaling pathways that bypass cell cycle checkpoints (14).

Emerging clinical evidence highlights the pivotal role of genetically determined microbial alterations in destabilizing gut-liver axis, which accelerates steatohepatitis progression, exacerbates fibrogenesis, and ultimately fosters hepatocarcinogenesis (25–27). The pathogenic interplay between intestinal inflammation, gut microbiota alterations, and cholangiopathies is strikingly exemplified in PSC (28). In genetically predisposed individuals, gut-derived microbial components trigger cholangiocyte-specific immune activation, initiating or perpetuating biliary epithelial injury (29). Furthermore, gut dysbiosis critically impairs antitumor immune surveillance and propels the transition from chronic biliary disease to malignancy by orchestrating a pro-inflammatory microenvironment (19).

## 3 Gut microbiota and cholangiocarcinoma development

### 3.1 Gut microbiota and cholangiocarcinoma carcinogenesis

CCA patients exhibit significant gut microbiota dysbiosis, characterized by reduced microbial diversity, depletion of beneficial taxa (e.g., *Faecalibacterium*), and enrichment of pathobionts (e.g., *Escherichia-Shigella*), which collectively disrupt gut-liver axis homeostasis (**Table 1**). Of note, α-diversity alterations exhibit marked heterogeneity across CCA subtypes, with conflicting reports in extrahepatic and hilar variants (30). Emerging evidence on iCCA consistently demonstrate microbial dysbiosis accompanied by reduced α-diversity, likely reflecting tumor microenvironment-driven ecological pressures. In a study by Zhang et al., it was revealed that patients with iCCA exhibited significantly reduced abundances of beneficial gut bacteria *Bacteroides*, *Faecalibacterium*, and *Roseburia*, along with increased levels of pathogenic taxa *Escherichia-Shigella* and *Subdoligranulum*, compared to healthy controls (16). This study firstly unveiled that gut microbiota modulates glutamine metabolism to downregulate the ALK5/NOX1 axis, thereby inhibiting ferroptosis in CCA cells, offering a promising therapeutic strategy for precision treatment in iCCA. Moreover, Ito et al. reported enrichment of *Enterobacteriaceae* (harbouring the polyketide synthase carcinogenic island) in biliary tract cancer (BTC) faecal samples, suggesting its direct pro-carcinogenic role in biliary tract carcinogenesis (31). A case-control study and a cohort analysis found no significant difference in gut microbiota α-diversity between CCA patients and healthy controls (21, 32). Notably, within this cohort study, a machine learning-driven approach leveraging a random forest algorithm identified eight discriminative bacterial genera (e.g., *Faecalibacterium*, *Klebsiella*) to develop a tripartite microbial signature-based classifier, achieving exceptional diagnostic accuracy (AUC: 0.92–0.99) in effectively differentiating CCA from HCC (21). Furthermore, CCA patients exhibited increased abundance of Gram-negative bacteria (*Enterobacteriaceae*), correlating with elevated systemic inflammation markers (neutrophil-to-lymphocyte ratio, platelet-to-lymphocyte ratio). In contrast, Jia et al. revealed elevated α-diversity patterns in iCCA patients through a multi-omics approach integrating taxon-specific microbial biomarkers (notably *Lactobacillus* and *Alloscardovia*) with functional BA metabolism signatures (33). Despite established links between specific gut microbiota alterations​​ (e.g., decreased *Faecalibacterium*, increased *Escherichia-Shigella/Enterobacteriaceae*) ​​and CCA pathogenesis​​ (16, 31, 33), ​​interpretation remains constrained by methodological limitations. Predominantly small cohort sizes​​ compromise statistical power and hinder validation/generalizability of findings, including biomarker classifiers or subtype-specific diversity patterns (e.g., the α-diversity heterogeneity observed) (16, 21, 31–33). ​​Furthermore, reliance on observational designs​​ impedes causal inference due to uncontrolled, dynamic confounders such as recent antibiotic use, dietary fluctuations, and variable underlying liver pathology (e.g., cirrhosis severity). Consequently, ​​attributing dysbiosis directly to CCA versus confounders/disease consequences remains challenging.​​ Future research necessitates ​​large-scale, multi-center prospective cohorts​​ with ​​rigorous longitudinal monitoring of key confounders (antibiotics, diet, liver function)​​ and ​​robust causal inference methods.​.

**Table 1:** Gut microbiota alterations in cholangiocarcinoma.

Reference	Gut microbiota	Groups	Method	α-diversity	Association with development	Study limitations
(16) Zhang Q	Pathogenic: *Escherichia-Shigella* *↑* *Subdoligranulum ↑*	iCCA/healthy	Metabolomics, 16S rRNA sequencing	↓	Glutamine inhibits ferroptosis via the ALK5/NOX1 axis, promoting tumor growth.	Animal-human model discrepancies; Mechanistic details unclear.
	Protective: *Bacteroides*↓ ***Faecalibacterium*↓** *Roseburia*↓				
(31) Ito Z	Pathogenic: ​​ ***Enterobacteriaceae*** *↑*	BTC/BBD/healthy	16S rRNA sequencing	↓	*Enterobacteriaceae​​* (harboring pks gene): promotes biliary carcinogenesis.	Small sample size; Observational studies cannot exclude confounders (e.g., antibiotic use).
	Protective: *Faecalibacterium*↓​​*Coprococcus​​*↓					
(21) Deng T	Pathogenic: Gram-negative bacteria (e.g., ***Enterobacteriaceae*** ↑)	CCA/HCC/healthy	16S rRNA sequencing	No difference	Gram-negative bacteria correlate with inflammatory markers (NLR, PLR); Beneficial genera reduction linked to poor prognosis.	Confounding factors (diet, medications) not controlled.
	Protective: ***Faecalibacterium*** ↓					
(32) Zhang T	Pathogenic: *genera Bacteroides*↑ *Muribaculaceae_unclassified*↑ *Muribaculum*↑ *Alistipes*↑	CCA/choleithiasis/healthy	16S rRNA sequencing	No difference	*General Burkholderia-Caballeronia-Paraburkholderia*, *Faecalibacterium*, and *Ruminococcus_1* for CCA early diagnosis.	Small sample size.
	Protective: NA					
(33) Jia X	Pathogenic: *Lactobacillus* ↑ *Actinomyces* ↑ *Peptostreptococcaceae* ↑ *Alloscardovia* ↑ *Ruminococcaceae* ↑	iCCA/HCC/cirrhosis/healthy	16S rRNA sequencing, Mass spectrometry for bile acids	↑	*Ruminococcaceae* enrichment in vascular invasion; ​​ ​​*Pseudoramibacter​​* is negatively correlated with survival time	Small sample size; Mechanisms unexplored.
	Protective: NA					
(34) Zhang Y	Pathogenic: *Eubacterium hallii group* ↑ *Candidatus Soleaferrea* ↑ *Flavonifractor* ↑	BTC/HCC	MiBioGen + IEU GWAS	NA	Associated with increased BTC risk and tumorigenesis via inflammation or metabolic pathways.	Data from diverse regions/ethnicities may introduce bias.
	Protective: *Dorea* ↓ *Lachnospiraceae ND3007 group* ↓				May suppress tumor progression by modulating secondary bile acids or anti-inflammatory effects.	
(35) Chen Z	Pathogenic: *Veillonellaceae* ↑ *Alistipes*↑ ***Enterobacteriales↑*** *Firmicutes* ↑	iCCA	MiBioGen + IEU GWAS	NA	May promote ICC via endotoxin production or activation of pro-tumor pathways (e.g., AMPK/mTOR).	GWAS data primarily from European populations limits generalizability. Lack of mechanistic validation.
	Protective: *Anaerostipes*↑ *Paraprevotella*↑ *Parasutterella*↑ V*errucomicrobia*↑				May enhance intestinal barrier function (via butyrate) or suppress inflammation.	
(36) Zhang L	Pathogenic: *Candida* ↑ *C. albicans* ↑	iCCA/healthy	ITS2 rDNA sequencing	↓	*C. albicans* abundance increases in advanced TNM stages; *Saccharomyces cerevisiae* reduction linked to iCCA.	Small sample size; Fungal functional roles and host interactions not validated.
	Protective: *Saccharomyces cerevisiae* ↓					

The pathogenic bacterium ​​Enterobacteriaceae​​ showed an increase ​​in the findings of Ito Z et al., Deng T et al., and Chen Z et al.​​; the protective bacterium ​​Faecalibacterium​​ exhibited a decrease ​​in the results of both Zhang Q et al. and Deng T et al.​

Mendelian randomization analyses leveraging MiBioGen gut microbiota genome-wide association study (GWAS) data have identified host genetic variants causally associated with microbial compositional changes. These analyses revealed that elevated abundances of *Eubacterium hallii* group, *Candidatus Soleaferrea*, and *Flavonifractor* confer increased BTC risk, while *Dorea* and *Lachnospiraceae* ND3007 demonstrated protective effects (34). Complementary Mendelian randomization studies further implicated *Veillonellaceae*, *Alistipes*, *Enterobacteriales*, and *Firmicutes* as iCCA-promoting taxa (35). Mechanistically, protective genera such as *Anaerostipes*, *Paraprevotella*, *Parasutterella*, and *Verrucomicrobia* enhance intestinal barrier integrity via butyrate production or exert anti-inflammatory activity, underscoring the dual diagnostic and therapeutic potential of gut microbiota in hepatobiliary malignancies. Notably, iCCA patients demonstrate gut mycobiota dysbiosis characterized by *Candida* spp. (particularly *C. albicans*) enrichment and depletion of beneficial *Saccharomyces cerevisiae*, with advanced TNM stages (III–IV) correlating strongly with pathogenic fungal predominance (36). These findings position gut microbiota dysbiosis as a driver of iCCA progression, warranting exploration of antifungal therapies (**Supplementary Figure 1**). However, critical gaps persist in addressing bacterial-fungal ecological interactions central to dysbiosis mechanisms and establishing experimental validation of causality between specific fungal taxa (e.g., *C. albicans*) and oncogenesis through animal models or ***in vitro*** systems.

While emerging evidence reveals subtype-specific gut microbial configurations differentiating CCA and healthy controls, current investigations remain constrained by geographically restricted cohorts. Future studies should prioritize mechanistic validation through germ-free animal models ​​integrated with​​ multi-ethnic validation cohorts—​​stratified into three principal groups​​: (1) Southeast Asian cohorts (≥40% allocation, bearing >50% of the global CCA burden), (2) European/North American cohorts (addressing industrial/environmental confounders), and (3) latent-risk diaspora cohorts. Crucially, ancestry-based principal component analysis with structured recruitment quotas must mitigate cryptic population substructure and allelic sampling biases, ​​thereby ensuring robust control​​ of geographic and migrant heterogeneity. ​​This integrated methodology​​ will enable rigorous delineation of causal host-microbiota interactions while controlling population stratification artifacts.

### 3.2 Gut microbiota and cholangiocarcinoma prognosis

Emerging evidence positions the gut microbiota as a pivotal determinant of CCA progression through multifaceted molecular mechanisms. Prognostically, tumor vascular invasion (VI) shows strong correlations with specific gut microbial alterations (37) (**Supplementary Figure 1**). VI-positive patients exhibit distinct dysbiotic patterns marked by *Oscillospiraceae* enrichment and depletion of beneficial taxa (*Eubacteriaceae, Allobaculum, Pediococcus*), patterns that independently predict reduced survival and elevated recurrence risks (33). Notably, advanced TNM stages (III-IV) in intrahepatic CCA show marked *Candida albicans* overgrowth in gut mycobiota, suggesting fungal dysbiosis as a potential progression biomarker (36). The microbial-immune interface further reveals VI-associated immunological shifts, with *Ruminococcaceae*-enriched cases displaying elevated IL-4 and suppressed IL-6 levels (21), highlighting microbiota-driven immune microenvironment remodeling.

Therapeutically, gut microbial signatures demonstrate predictive value for treatment responses across modalities. While *Bacteroidetes* enrichment (particularly *Alistipes* sp. *Marseille-*P5997) associates with improved anti-programmed cell death protein 1(PD-1) immunotherapy outcomes in biliary tract cancers (38), Proteobacteria dominance inversely correlates with Sintilimab-anlotinib combination efficacy in advanced cases (39). Mechanistically, microbial metabolites like cyclic dinucleotide c-di-AMP enhance radiotherapy-induced antitumor immunity through STING pathway activation (40), revealing novel microbiome-mediated therapeutic sensitization strategies. Nevertheless, critical knowledge gaps persist in delineating precise microbiota-immune crosstalk mechanisms and validating microbial biomarkers for clinical translation. Systematic investigation of host-microbiota metabolic interactions and standardized multi-omics approaches remain imperative to harness the full therapeutic potential of gut microbiome in CCA management.

### 3.3 Gut microbiota-derived metabolites and cholangiocarcinoma

The intestinal barrier serves as a critical interface that maintains microbial homeostasis through selective permeability, effectively restricting the translocation of exogenous and microbiota-derived molecules under physiological conditions (41). While inherently plastic adapts to physiological demands, this barrier exhibits vulnerability to age-related deterioration, environmental stressors, and pathological insults. Gut microbiota-derived metabolites, including those directly synthesized by bacteria or enzymatically transformed from dietary and host-derived molecules, exert oncogenic effects via bidirectional crosstalk with host signaling pathways. Dysbiosis disrupts intestinal barrier integrity, enabling systemic translocation of microbial components, including structural molecules (e.g., lipopolysaccharides, LPS) and metabolic byproducts (e.g., short-chain fatty acids (SCFAs)) (42–44). These translocated products, particularly endotoxins, access the liver via portal circulation, activating hepatic Kupffer cells (KCs) to initiate pro-inflammatory cytokine cascades (e.g., tumor factor alpha [TNF-α], IL-6) that perpetuate chronic inflammation, bile duct injury, and metabolic dysregulation (45, 46).

Elevated circulating LPS, a hallmark of Gram-negative bacterial dysbiosis, is clinically associated with chronic hepatobiliary diseases and malignancy progression. Impaired tight junction integrity (e.g., reduced Claudin-1/Occludin expression) facilitates LPS translocation to the liver (47). where it drives tumorigenesis via TLR4-mediated activation of oncogenic pathways (e.g., NF-κB, PI3K/AKT) (48, 49). Diet-induced metabolic perturbations exacerbate this cycle by promoting barrier dysfunction and endotoxemia, as evidenced in nonalcoholic fatty liver disease models (50, 51). In PSC models, dysbiosis-driven LPS translocation activates hepatic TLR4/MyD88 signaling, exacerbating cholangitis and fibrosis to establish a pro-carcinogenic niche (52). Patients with iCCA exhibit gut microbiota shifts marked by opportunistic pathogen enrichment (e.g., *Veillonella, Klebsiella)*, with pathogen-derived LPS activating TLR4 on cholangiocytes and KCs to induce NF-κB-driven IL-6/TNF-α production, amplifying biliary injury and cholestasis (21). Mechanistically, LPS enhances tumor aggressiveness via METTL3-mediated PI3K/AKT pathway activation, promoting tumor cell migration and invasion (17). Additionally, LPS orchestrates immunosuppression in the tumor microenvironment (TME) by triggering TLR4-dependent hepatocyte C-X-C motif chemokine ligand (CXCL)1 secretion, which recruits polymorphonuclear myeloid-derived suppressor cells (PMN-MDSCs) to dampen antitumor immunity (19). These findings collectively position the LPS/TLR4 axis as a therapeutic target, suggesting microbiota modulation and microenvironment reprogramming as viable strategies to combat iCCA progression.

### 3.4 Biliary microbiota and cholangiocarcinoma development

The biliary system harbors gut-associated microbes including *Escherichia coli* and *Enterobacter* spp., translocated via the gut-liver axis or establish local colonization (53). Systematic characterization by Carlo et al. revealed that biliary enrichment of *E. coli* and *Klebsiella pneumoniae* serves as an independent prognostic marker for reduced survival in pancreaticobiliary malignancies, highlighting their potential as diagnostic biomarkers (54). Notably, *Alcaligenes faecalis* demonstrates TME-specific colonization in eCCA, exhibiting diagnostic potential for early tumor detection and therapeutic monitoring (55). Contrasting with cholangitis-associated microbiotas, perihilar CCA exhibits a Gram-positive predominance featuring *Enterococcus* spp., which independently predicts portal vein thrombosis and multidrug resistance development (56). Furthermore, biliary colonization by *Enterococcus* or *Candida* spp. correlates strongly with clinical progression in PSC, solidifying microbial dysbiosis as a prognostic determinant (57). Critically, antibiotic-resistant strains such as *Enterococcus faecalis* and *Enterobacter* spp. in hepatic ducts directly exacerbate postoperative infection rates and mortality in eCCA, underscoring the clinical imperative for microbiota-guided perioperative management (58). These findings collectively establish biliary microbiota as both a driver of oncogenesis and a therapeutic target in CCA progression.

### 3.5 Oral microbiota and cholangiocarcinoma development

Comprising approximately 770 phylogenetically diverse species, the oral microbiota emerges as a pivotal regulator of systemic immunity and metabolic homeostasis with broad disease implications (59, 60). The functional crosstalk between oral and gut microbiotas has gained prominence given their collective influence on human pathophysiology (61), particularly through pathobiont translocation along the gut-liver axis during dysbiosis (62). Clinically, Rao and colleagues developed an oral microbiota-based diagnostic algorithm incorporating three bacterial biomarkers (*Lautropia*, *Alloprevotella*, and *Actinomyces)*, achieving exceptional accuracy (AUC=0.981) in differentiating iCCA from HCC (63). A complementary Korean cohort study further demonstrated stratified oral microbial signatures across upper gastrointestinal malignancies, revealing significantly elevated α-diversity in oesophageal/gastric cancers compared to BTC and pancreatic cancer cohorts (64). Intriguingly, while distinct diagnostic microbial profiles were identified for oesophageal/gastric malignancies, BTC and pancreatic cancers lacked comparable signatures. This disparity suggests an anatomic microbial gradient along the gastrointestinal tract, where tumor proximity to the oral cavity correlates positively with both microbial dysbiosis severity and diagnostic biomarker detectability. While BTC-specific oral microbial biomarkers remain unidentified, these findings provide foundational evidence for the oncogenic involvement of the oral-gut-liver axis in BTC pathogenesis. Subsequent research must prioritize multi-institutional cohorts with longitudinal sampling to resolve spatiotemporal dynamics of microbial translocation and enable mechanistic dissection of tumor-promoting crosstalk within this axis.

## 4 Bile acid dysregulation in cholangiocarcinoma

The enterohepatic circulation maintains BA homeostasis through a hepatobiliary-intestinal loop, wherein hepatocyte-derived BAs undergo intestinal reabsorption followed by portal venous return to the liver (65, 66). Intrahepatic BA overload, while non-carcinogenic, drives cholangiocarcinogenesis via ductular hyperplasia, inflammatory niche formation, and impaired cytoprotective BA signaling (67). CCA development is strongly linked to chronic cholestasis, implicating prolonged exposure to elevated BA levels in promoting gastrointestinal carcinogenesis. Emerging evidence highlights BA metabolic reprogramming as a critical determinant of CCA heterogeneity and progression. The following will examine alterations in BA profiles in CCA, elucidates mechanisms through which BAs drive CCA pathogenesis, and evaluates their clinical implications.

### 4.1 Changes in bile acid profiles in cholangiocarcinoma

Serum total bile acid levels progressively escalate across the disease continuum from healthy controls to benign biliary disease (BBD) and CCA patients, correlating with biliary obstruction severity (68). Conjugated bile acids exhibit marked accumulation, particularly primary conjugated BAs (glycocholic acid [GCA], taurochenodeoxycholic acid [TCDCA]) showing marked increases in both serum and bile compared to BBD and healthy controls (69, 70) (**Table 2**). Paradoxically, secondary conjugated species (glycolithocholic acid [GLCA], glycoursodeoxycholic acid [GUDCA]) demonstrate malignant-specific reduction (71). Concurrently, primary unconjugated BAs (cholic acid [CA], chenodeoxycholic acid [CDCA]) accumulate due to cholestasis-induced excretion impairment (69), whereas secondary unconjugated derivatives (deoxycholic acid [DCA], lithocholic acid [LCA]) decline in plasma, reflecting gut microbial 7α-dehydroxylation defects (72). This conjugated/unconjugated BA ratio imbalance strongly correlates with suppressed bacterial deconjugation activity, establishing a pathogenic feedback loop between hepatic BA synthesis and gut microbial metabolic reprogramming that drives carcinogenesis. Thus, between-study heterogeneity in BA levels (particularly CCA vs BBD discrepancies) arises from methodological variables including biospecimen type (serum/bile/tissue), cohort heterogeneity, and gut microbiota-mediated BA metabolic variation. Critically, current BA metabolomic validation in CCA faces three critical gaps: (1) ​​absence of multi-center harmonization​​ in pre-analytical (sample collection/storage) and analytical (LC-MS) workflows; (2) ​​deficient dynamic monitoring​​ failing to track tumor progression/therapy-induced BA flux changes; and (3) ​​inadequate pathological specificity​​ against cirrhosis/cholangitis confounders. These limitations mandate prospective longitudinal cohorts with protocolized multi-omics integration to resolve CCA-specific BA dynamics.

**Table 2:** Dysregulation of bile acid metabolism in cholangiocarcinoma.

​Reference	​Study population	Analytical methods	​BA metabolic alterations	​Clinical relevance
(69) Proungvitaya S	- CCA patients (n=10) - Benign biliary disease (BBD) patients (n=9) - Healthy controls (n=8)	HPLC (9 BAs)	- Elevated total serum BA in CCA - Glycocholic acid (GCA) increased in CCA/BBD (non-significant) - Distinct BA profiles in CCA vs. BBD/controls	- GCA as a potential biomarker - BA profiling aids CCA diagnosis but requires validation
(70) Zhang X	- CCA (n=42) - BBD (n=120) - Healthy controls (n=120) - Gallbladder cancer (GC)/HCC controls	UPLC-MS/M (15 BAs)	- Elevated conjugated BAs (TCDCA) in CCA - Reduced unconjugated BAs (CDCA) - Imbalanced conjugated/unconjugated BA ratio	- CDCA + TCDCA panel outperformed CA19-9 (AUC=0.95) - BA profiles distinguish CCA from HCC/GC
​ (71) Rejchrt S	Benign(n=7) malignant biliary strictures (n=21); non-stenotic controls (n=45)	HPLC/MS (23 BAs)	-Reduced GLCA, GUDCA in malignant group - Significant CA variation between benign/malignant groups	- Serum total bilirubin elevated in malignancy - CA as diagnostic discriminator
​ (72) Wang W	CCA patients (n=36); Healthy volunteers (n=35)	TCGA data analysis Metabolomics	- 15 differential BAs (e.g., HDCA, isoLCA) - Downregulated primary BA biosynthesis and secretion	- Diagnostic panel (HDCA/isoLCA/bCDCA/DCA): Sensitivity: 0.933; Specificity: 0.867
​ (86) Deng M	CCA patients (n=13); healthy controls (n=16); validation cohorts(FU-CCA, GSE89749)	-UPLC-MS/MS(30 BAs) -Single-cell RNA sequencing - Consensus clustering	- Elevated primary BAs; reduced secondary BAs - BA metabolic subtypes: active vs. inactive	- Active subtype: Poor prognosis, immunotherapy resistance - Prognostic markers: SLCO1B3, CEACAM1
(87) Farhat Z	- Hepatocellular carcinoma (n=201) - Fatal liver disease (n=261) - Biliary tract cancer (n=138) - Healthy controls	LC-MS/MS (15 BAs)	- Conjugated primary BAs (TCA, TCDCA, GCA) strongly linked to liver disease risk - Weak associations with secondary BAs (DCA, LCA)	- Conjugated BAs (e.g., TCA, TCDCA) associated with liver mortality (OR=4.1–9.7) - No significant link to biliary tract cancer

### 4.2 Mechanisms of bile acid dysregulation driving CCA

**Figure 2:**
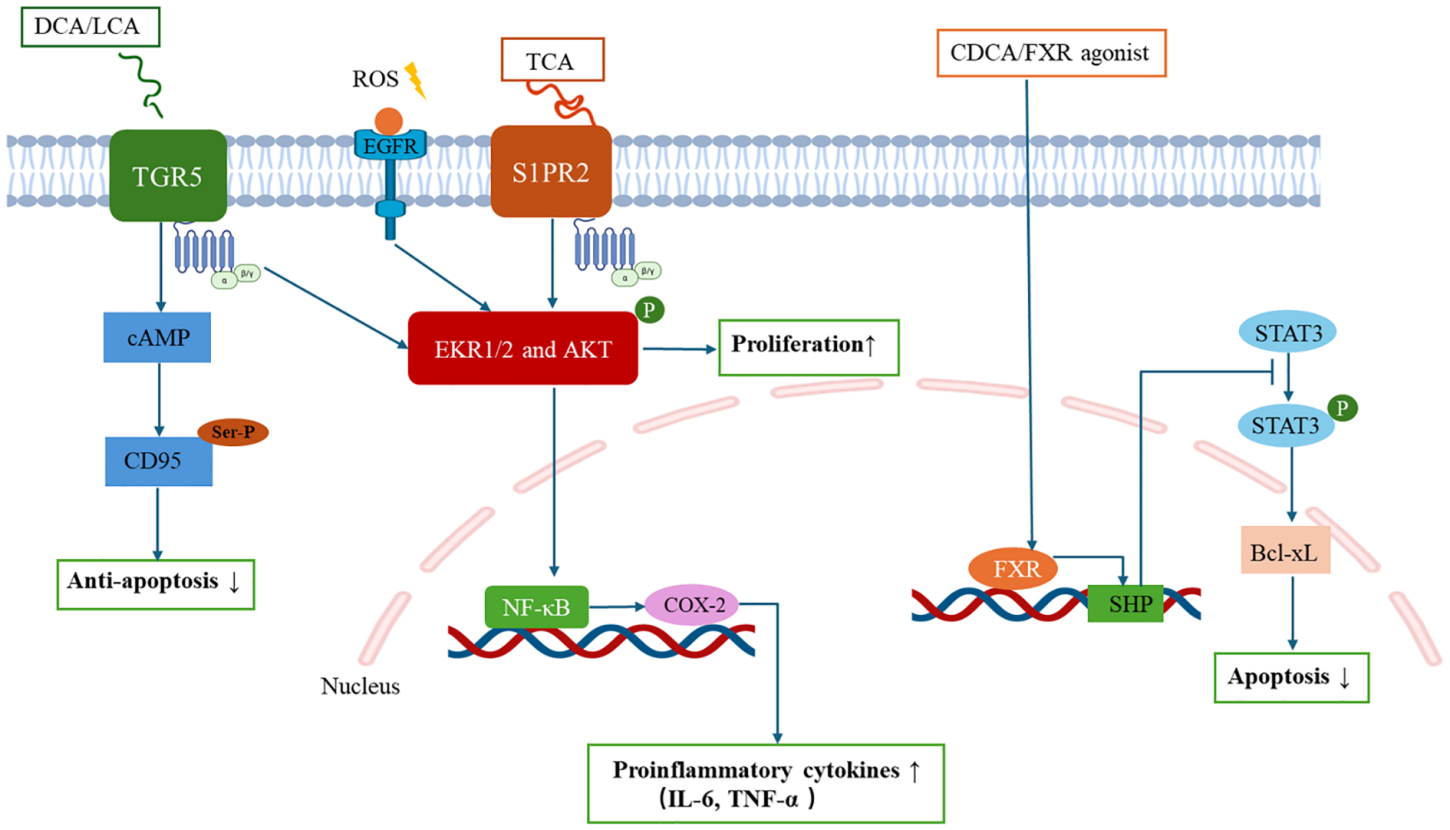
Bile acid receptor-mediated intracellular signaling pathways in cholangiocarcinoma cells.

Cholestasis-induced BA overload initiates a self-perpetuating pathogenic cycle central to CCA progression. Excessive BAs activate hepatocyte-cholangiocyte transdifferentiation via dual receptor systems: nuclear receptors (farnesoid X receptor, FXR) and membrane-bound receptors (Takeda G protein-coupled receptor 5, TGR5; sphingosine-1-phosphate receptor 2, S1PR2) (73, 74) (**Figure 2**). Sustained BA-receptor engagement generates a pro-tumorigenic niche marked by oxidative DNA damage (via reactive oxygen species [ROS] overproduction) and apoptotic resistance (through CD95 inactivation), thereby completing malignant transformation. This BA signalling duality manifests through distinct pathways: (1) Nuclear receptor modulation: Although frequently downregulated in CCA (84), FXR activation by CDCA or synthetic agonists (e.g., GW4064) exerts tumor-suppressive effects (75, 76). Mechanistically, FXR activation induces small heterodimer partner (SHP) expression, which suppresses STAT3 phosphorylation to downregulate Bcl-xL in BTC cells, ultimately triggering apoptosis and inhibiting proliferation (77–79). (2) Membrane receptor cascades: TGR5 promotes cholangiocyte proliferation through extracellular signal-regulated kinase (ERK)1/2 phosphorylation via a cascade comprising ROS generation, Src kinase activation, and epidermal growth factor receptor (EGFR) transactivation. Simultaneously, TGR5 triggers PKA-mediated CD95 serine/threonine phosphorylation that blocks death receptor signalling, conferring apoptotic resistance, with these dual mechanisms synergistically driving cholangiocarcinogenesis (80–82). S1PR2 axis: TCA-mediated S1PR2 activation triggers ERK1/2/Akt signalling through both G protein-dependent and EGFR-mediated transactivation pathways, subsequently inducing NF-κB activation and cyclooxygenase-2 (COX-2) upregulation. This signalling axis coordinately upregulates proinflammatory cytokines (IL-6, TNF-α), thereby establishing a self-sustaining inflammatory niche that promotes CCA progression (83–85).

BA signalling orchestrates TME immunosuppression and metastatic priming through macrophage polarization and T-cell functional suppression. S1PR2 activation by conjugated BAs upregulates COX-2/PGE2 signalling, which directly suppresses CD8+ cytotoxic T lymphocyte activity while promoting regulatory T cell infiltration, thereby establishing an immune-evasion niche conducive to tumor progression (83, 84). The functional duality of TGR5 pivots on ​​disease stage-specific microenvironmental cues​​. In ​​cholestatic conditions​​ (e.g., early-stage PSC), TGR5 activation exerts hepatoprotective effects by mediating M2 macrophage polarization and suppressing NF-κB-mediated inflammation​​ via cAMP-PKA signalling (52). Conversely, within the ​​established TME of advanced CCA, TGR5 signalling shifts toward pro-tumorigenic activity by conferring apoptosis resistance and enhancing proliferation in malignant transformed biliary epithelial cells (81). The precise molecular mechanisms and pathological staging thresholds governing the transition of TGR5 from hepatoprotective to protumorigenic function remain incompletely characterized. Current evidence suggests this transition may correlate with ​​progression of biliary intraepithelial neoplasia, ​​advanced cirrhotic remodelling​​, ​​sustained BA accumulation​​, and ​​immune reprogramming. This receptor dichotomy underscores the delicate equilibrium between BA-mediated immune regulation and malignant transformation in hepatobiliary diseases. Elucidating these context-dependent switches represents a critical research priority for developing stage-adapted TGR5-targeted therapies in CCA.

### 4.3 Clinical significance of bile acid dysregulation in CCA

Clinical evidence highlights the critical association between dysregulated BA metabolism and the diagnosis, prognostic evaluation, and therapeutic strategies for CCA. BA profiling has emerged as a potential source of non-invasive biomarkers for CCA diagnosis (**Table 2**). Zhang et al. (70), developed a diagnostic model based on CDCA and TCDCA, which effectively distinguished CCA from BBD, gallbladder cancer, and HCC, demonstrating significantly superior specificity compared to the conventional biomarker CA19-9. Multi-omics studies further identified a quadruple diagnostic panel comprising hyodeoxycholic acid, isoLCA, bCDCA, and DCA, achieving 93.3% sensitivity and 86.7% specificity (72). Notably, serum GCA exhibits diagnostic potential but shows limited specificity in differentiating malignant from benign biliary disorders (69).

Metabolomic stratification studies demonstrate that BA metabolic activity serves as an independent prognostic determinant in CCA. Molecular subtyping based on BA metabolism features classifies CCA into metabolically active and inactive subgroups, where the active subtype exhibits significantly shorter OS and impaired immunotherapy response (86). These findings establish BA metabolic reprogramming as a mechanistic basis for precision oncology, providing a molecular classification framework for personalized therapeutic strategies. Intriguingly, while conjugated primary BAs (e.g., TCA, TCDCA) demonstrate strong correlation with HCC mortality, this association remains absent in BTCs, suggesting tumor-specific metabolic rewiring mechanisms (87). Overcoming these challenges necessitates collaborative multicenter studies that integrate machine learning pipelines to discriminate disease-relevant metabolic signatures from inter-individual physiological variability.

## 5 Gut microbiota-bile acid crosstalk in cholangiocarcinogenesis

**Figure 3:**
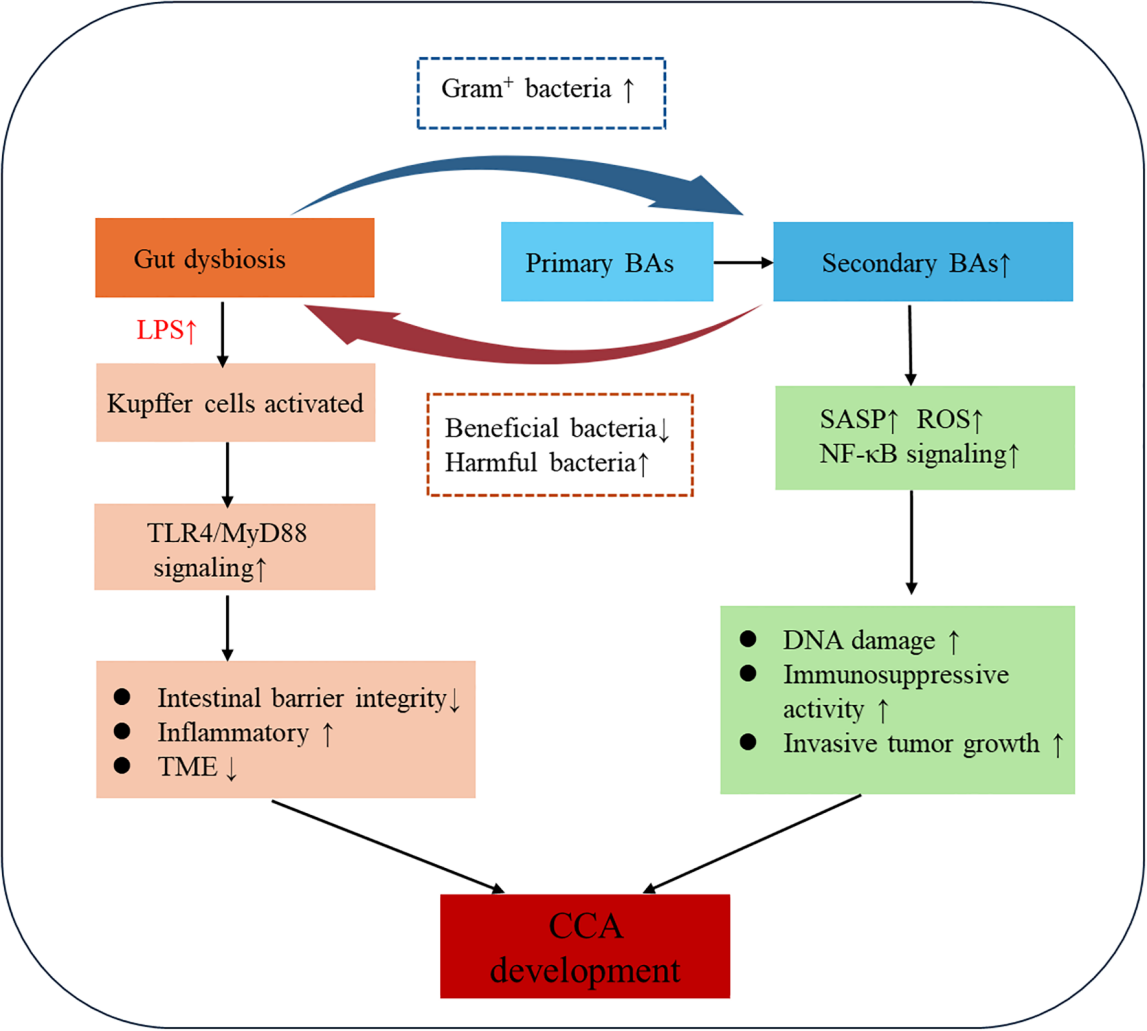
Mechanistic diagram of bile acid-microbiome-immune crosstalk in cholangiocarcinoma.

The gut-liver axis establishes a bidirectional regulatory network wherein microbial BA metabolism and BA-mediated microbiota modulation cooperatively drive CCA progression through integrated metabolic and immune signaling (88). Gut commensals enzymatically transform primary BAs into secondary species (e.g., deconjugation, 7α-dehydroxylation), which in turn regulate microbial ecology and activate tumor-promoting pathways via receptor-mediated mechanisms (89, 90). This bidirectional crosstalk drives cholangiocarcinogenesis through chronic biliary inflammation, epigenetic reprogramming of cholangiocytes, and TME remodeling (**Figure 3**).

### 5.1 Microbial regulation of BA metabolism in CCA pathogenesis

Gut microbiota-derived enzymes convert primary BAs to tumorigenic secondary species (DCA, LCA) that activate oncogenic signalling cascades (91, 92). Clostridium scindens and other 7α-dehydroxylase-expressing species generate secondary BAs associated with diet-induced hepatocarcinogenesis models, where dysbiosis elevates circulating DCA. This hydrophobic secondary BA drives tumorigenesis by inducing senescence-associated secretory phenotype activation, ROS overproduction, and NF-κB-mediated inflammation via gut barrier disruption (93). Notably, DCA impairs hepatic CXCL16 secretion in liver sinusoidal endothelial cells, reducing CXCR6+ natural killer cell (NKT) recruitment and compromising antitumor immunity. Pharmacological depletion of Gram-positive 7α-dehydroxylase bacteria (e.g., vancomycin) attenuates secondary BA production and exerts chemopreventive effects in preclinical models (94). Furthermore, microbial bile salt hydrolases dynamically remodel the BA pool, facilitating intestinal absorption or further modification of BA (95). Therapeutic microbiota modulation demonstrates dual mechanisms: (1) *Lactobacillus rhamnosus* GG (LGG) activates FXR to reduce BA accumulation in cholestatic liver disease (96, 97); (2) *Pediococcus pentosaceus* Li05 enhances bile salt hydrolase activity while suppressing NLRP3 inflammasome activation in PSC (98, 99). These findings position gut microbiota as master regulators of BA homeostasis, linking microbial ecology to inflammation-driven carcinogenesis.

### 5.2 BA-mediated control of gut microbial dynamics in CCA

BAs critically shape gut microbiota composition and homeostasis through their chemical properties and signalling functions (67, 100). Primary BAs (e.g., CA, CDCA) exhibit surfactant-like properties that disrupt bacterial cell membrane integrity and interfere with DNA stability, thereby inhibiting pathogens such as *Clostridium difficile*, *Salmonella* spp., and *Listeria* spp. Notably, Gram-positive bacteria (*Firmicutes*) demonstrate heightened susceptibility to these antimicrobial effects compared to Gram-negative counterparts, a phenomenon attributable to structural differences in their cell wall architectures (101). Moreover, gut microbiota converts primary BAs into secondary BAs (e.g., DCA) through bile salt hydrolase and 7α-dehydroxylase activities. This biotransformation not only modulates BA toxicity and signalling activity but also reshapes microbial ecology via metabolic crosstalk. DCA selectively inhibits beneficial bacteria (e.g., *Lactobacillus)* while promoting the expansion of pathobionts such as *Enterobacteriaceae*, fostering a pro-inflammatory microenvironment (22).

Furthermore, BA signalling orchestrates gut microbial homeostasis through BA receptor-mediated pathways, predominantly FXR. Mechanistically, FXR activation stimulates the expression of antimicrobial peptides such as angiogenin-1, effectively curbing the expansion of pathobionts within the Proteobacteria phylum (102), while concurrently fortifying intestinal barrier function by upregulating tight junction proteins (e.g., occludin, ZO-1) (103). This dual regulatory axis not only maintains microbial equilibrium but also mitigates systemic inflammation triggered by bacterial endotoxin leakage. Future studies should delineate the mechanisms through which BA pool-mediated microbiota modulation governs host-microbial interactions.

## 6 Therapeutic translation: targeting the gut-liver axis in clinical practice

Gemcitabine/cisplatin combination therapy maintains its position as the standard first-line chemotherapeutic regimen for CCA, yet persistent limitations in objective response rates continue to challenge clinical outcomes. Emerging adjuvant strategies targeting the gut-liver axis show therapeutic promise, particularly given the pivotal role of gut microbiota dysbiosis in CCA pathogenesis. BA metabolism imbalance in specific CCA subtypes reveals a treatable weakness, positioning FXR/TGR5-targeted therapies as tailored approaches to fix cancer-driven metabolic defects in these patient groups. Therapeutically targeting this microbiota-metabolite-immune triad holds promise for intercepting CCA progression, advancing precision medicine through multimodal pathway modulation in CCA management (**Table 3**).

**Table 3:** Therapeutic strategies targeting the gut-liver axis in cholangiocarcinoma: molecular targets and mechanisms of action.

Category	Specific agents	Primary target	Core mechanism	Pathway/Action
Antibiotics	Vancomycin	Gram-positive bacteria	Depletion of BA-converting bacteria; TH17 suppression	↑ CXCL16-dependent NKT infiltration; ↓ Hepatic inflammation
	Neomycin	Gram-negative bacteria	LPS reduction; CXCL1-PMN-MDSC axis blockade	↓ Bacterial translocation; ↓ MDSC-mediated immunosuppression
Probiotics	*Lactiplantibacillus* spp.	Gut microbiota composition	FXR-FGF15 axis activation; Metabolite secretion	↓ BA synthesis; ↑ BA excretion; Tumor apoptosis via peptidoglycans
	*Pediococcus pentosaceus*Li05	BA homeostasis	FXR-dependent signaling restoration	Attenuates hepatic fibrosis; Anti-inflammatory effects
FMT	Healthy donor microbiota	Microbial dysbiosis	Microbial diversity restoration; Barrier integrity reinforcement	↓ Serum ALP; Reprograms microbiota-NET axis to reduce metastasis
BA Modulators	FXR Agonists (OCA)	FXR nuclear receptor	SHP/LRH-1 pathway activation	↓ CCA proliferation/migration; Synergizes with chemotherapy
	S1PR2 inhibitors (JTE-013)	S1PR2 receptor	ERK/AKT/NF-κB cascade inhibition	↓ Tumor invasiveness
	TGR5 inhibitors (SBI-115)	Tumor-associated macrophages	NF-κB/MAPK/ERK pathway suppression	↓ Pro-fibrotic and metastatic signalling cascades

### 6.1 Targeting gut microbiota dysbiosis

Targeted microbial modulation—via antibiotics, probiotics, or faecal microbiota transplantation (FMT)—represents a potential therapeutic strategy for CCA by restoring microbiota homeostasis (104) (**Table 4**). These interventions mechanistically counteract dysbiosis-driven oncogenesis through pathogen depletion, intestinal barrier restoration, and suppression of protumorigenic signalling. However, translational progress remains limited, with current evidence largely confined to preclinical models.

**Table 4:** Clinical trials of gut microbiota-targeted therapies for cholestatic liver disease.

Disease	Sample size (n)	Study type	Intervention	Outcomes	Status	NCT number	Reference
Antibiotics							
PSC	55	Single Group Assignment	Vancomycin: Oral 50mg/Kg per day up to a maximum of 1500 mg a day for three months.	Ten BA participants had surgery 1 week before starting the Vancomycin. On oral vancomycin, 9 PSC patients had improvement of LFTs, 8 had improvement of liver biopsies and/or MRI, and colon biopsies.	Completed	NCT01322386	
PSC and Biliary Atresia	59	Single Group Assignment	Vancomycin: Oral 50 mg/kg/day in 3 divided doses if weight <30kg, and 500 mg 3 times/day if weight ≥30kg.	Ninety-six percent, 81.3%, and 94.9% experienced a reduction of GGT, ALP, and ALT, respectively. Thirty-nine percent, 22%, and 55.9% experienced normalization of GGT, ALP, and ALT, respectively, within the first 6 months of OV treatment.	Completed	NCT01802073	
PSC and Biliary Atresia	200	Single Group Assignment	Oral Vancomycin	NA	Recruiting	NCT02137668	
PSC	28	Single Group Assignment	Vancomycin and amoxicillin: oral vancomycin (500 mg liquid twice a day) for 28 days and oral amoxicillin (1000 mg twice a day) for 7 days (during the last 7 days of the oral vancomycin).	NA	Recruiting	NCT06197308	
PSC	102	RCT	Oral Vancomycin	NA	Active, not recruiting	NCT03710122	
Probiotics							
Cholestatic liver disease	10	Case-Control study	Oral 1g fixed-dose compound Eosinophil-Lactobacillus tablets before three meals	*Lactobacillus acidophilus supplementat*ion ameliorates cholestatic liver injury	Completed	ChiCTR2200063330	110
FMT							
PSC	10	A pilot study	The patients underwent a single FMT (90 mL) on the right colon by colonoscopy.	FMT in PSC is safe and increases in bacterial diversity and engraftment correlates with an improvement in ALP.	Completed	NA	118
PSC	58	RCT	Filtered fecal suspension (0.6g/mL) aliquoted (50mL/tube). Colonoscopy: 150g stool in 250mL. Enema: 30g stool in 50mL, diluted to 100mL with 50mL normal saline.	NA	Recruiting	NCT06286709	119

#### 6.1.1 Antibiotics

Preclinical studies in PSC model reveal that gut barrier dysfunction and dysbiosis facilitate hepatic bacterial/endotoxin accumulation. *Klebsiella pneumoniae* exacerbates bacterial translocation and TH17-driven hepatic inflammation, whereas metronidazole or vancomycin suppresses TH17 activation, demonstrating microbiota-targeted therapeutic potential (105). Gut dysbiosis further induces hepatocyte-derived CXCL1 to recruit CXCR2+ PMN-MDSCs, accelerating CCA progression. Neomycin, a Gram-negative-specific antibiotic, blocks this CXCL1-PMN-MDSC axis, validating the gut-liver-CCA interplay linking microbial imbalance, barrier integrity, and antitumor immunity (19). Notably, vancomycin depletes Gram-positive bacteria that convert primary to secondary BAs, exerting antitumor effects in murine liver cancer models by enhancing CXCL16-dependent hepatic NKT cell infiltration and suppressing tumor growth.

Clinically, multiple trials (NCT01322386, NCT01802073, NCT02137668) report improved liver biochemistry in PSC patients treated with oral vancomycin (106). A randomized controlled trial (NCT03710122) further explored the immunomodulatory effects of vancomycin by analyzing pro-inflammatory cytokines (e.g., TGF-β, IL-4). A pilot study (NCT06197308) evaluates FMT combined with vancomycin/amoxicillin dual therapy for PSC. Despite preclinical efficacy, antibiotic-based strategies remain unexplored in human CCA, necessitating multicenter trials to assess safety and efficacy.

Paradoxically, while antibiotics targeting pathogenic bacteria may enhance PD-1 inhibitor efficacy in hepatobiliary cancers (107), prolonged antibiotic use correlates with reduced survival in HCC patients receiving anti-PD-1 therapy, likely due to microbiota disruption (108). Thus, cautious antibiotic administration—combined with probiotics or FMT—is critical to preserve microbiota balance. These findings underscore antibiotics as a dual-edged therapeutic strategy, with immunomodulatory effects mediated through precise gut-liver-immune axis regulation.

#### 6.1.2 Probiotics

Probiotic supplementation competitively colonizes gastrointestinal niches, mitigating intestinal dysbiosis by enhancing microbial homeostasis and inhibiting pathogenic bacterial adhesion. Emerging preclinical evidence highlights the therapeutic potential of *Lactiplantibacillus* species in CCA. For instance, LGG alleviates cholestasis by suppressing BA synthesis while enhancing excretion via activation of the intestinal FXR-FGF15 axis (96). Specific strains, including *Lactiplantibacillus plantarum* Lp 12 and Lp 355, exert dose-dependent antitumor effects against CCA by inducing tumor cell apoptosis and senescence through secreted metabolites such as peptidoglycans and exopolysaccharides (109). Synergistic interactions with gemcitabine further enhance antitumor responses while reducing chemotherapy doses and toxicity. Clinically, *Lactobacillus acidophilus* supplementation alleviates cholestatic liver injury through dual mechanisms: suppression of hepatic BA synthesis and enhancement of faecal BA excretion, as demonstrated in a registered case-control trial (ChiCTR2200063330) (110). Similarly, *Pediococcus pentosaceus* Li05 ameliorates PSC by activating the FXR-FGF15 axis, restoring BA homeostasis to attenuate hepatic inflammation and fibrosis (99). Traditional Chinese medicine formulations such as Si-Ni-San also modulate gut microbiota by promoting colonization of beneficial strains (e.g., *Parabacteroides goldsteinii*), thereby restoring microbial equilibrium and reinforcing intestinal barrier integrity (111).

Notably, probiotics may enhance immunotherapy efficacy. SCFAs derived from gut microbiota metabolism, including butyrate, exhibit anti-inflammatory and immunomodulatory properties. Specific taxa (e.g., *Lachnospiraceae, Ruminococcaceae*) enhance anti-PD-1 efficacy in BTC and HCC by mediating antitumor immunity through SCFAs and BA metabolic pathways (52). *Lactobacillus rhamnosus Probio*-M9 and *polyphenol castalagin* further potentiate anti-PD-1 responses by enriching immunotherapy-responsive bacterial taxa (e.g., *Ruminococcaceae, Alistipes*), offering strategies to overcome PD-1 resistance (112, 113).

Despite therapeutic promise, probiotics pose rare risks (114). Postoperative administration may lead to bacteraemia in immunocompromised patients, as reported in cases of *Clostridium butyricum*-associated bacteraemia following hepatic resection for biliary malignancies (115). Identified risk factors include intestinal barrier disruption, immunosuppression, and broad-spectrum antibiotic use. Clinicians should weigh benefits against potential adverse effects when prescribing probiotics. Collectively, microbiota-targeted interventions represent emerging strategies to optimize therapeutic outcomes in hepatobiliary cancers.

#### 6.1.3 FMT

FMT, which involves transferring processed donor faecal material to restore intestinal microbial balance (116), modulates gut-liver axis dysbiosis and represents a potential therapeutic strategy for cholestatic liver diseases (117). Preliminary clinical studies in PSC patients demonstrate FMT safety, with associated increases in gut microbial diversity and reductions in serum alkaline phosphatase levels (118). A phase IIa randomized trial (NCT06286709) assesses the therapeutic potential of repeated-colonic FMT in patients with PSC-associated inflammatory bowel disease. Key translational objectives include identifying mucosal multi-omics signatures (metagenomic, metatranscriptomic, metabolomic) and immunophenotypic pathways linked to FMT response, aiming to elucidate microbiota-host crosstalk mechanisms driving clinical outcomes (119). Gut dysbiosis promotes intrahepatic HCC metastasis through neutrophil extracellular trap hyperactivation, while healthy donor FMT prolongs survival and reduces tumor burden in murine models by reprogramming the microbiota-NET axis (120). These findings underscore the therapeutic potential of microbial interventions in hepatobiliary malignancies. However, both preclinical and clinical investigations into FMT for CCA management remain in their infancy, underscoring an urgent need for mechanistic and translational studies to address this critical knowledge gap.

### 6.2 Targeting bile acid mediated signalling pathway

Metabolic dysregulation, characterized by activation of BA, fatty acid, and xenobiotic pathways, represents a hallmark of CCA (121). Emerging strategies aim to disrupt BA-driven oncogenic networks while preserving metabolic homeostasis. S1PR2 inhibitors (e.g., JTE-013) suppress tumor invasiveness in CCA preclinical models by inhibiting ERK/AKT/NF-κB signalling cascades (85). Similarly, TGR5 activation in CCA cells promotes tumor progression, its downregulation in cholangiocytes and tumor-associated macrophages attenuates inflammation through NF-κB and MAPK/ERK pathway inhibition (52, 122). Intriguingly, TGR5 signalling exhibits ​​a stage-dependent duality​​: it ​​exerts hepatoprotective effects​​ during early cholestasis but ​​becomes pro-tumorigenic in established CCA (123). This functional switch necessitates carefully timed therapeutic interventions.​​ During the therapeutic window of biliary intraepithelial neoplasia, agonists​​ like INT-777 ​​may​​ ​​mitigate​​ malignant transformation ​​by restoring​​ anti-inflammatory and epithelial barrier functions. Conversely, in advanced CCA characterized by CAF-rich microenvironments, antagonists ​​such as​​ SBI-115 ​​can block​​ pro-fibrotic and pro-metastatic signalling cascades, ​​requiring stage-adapted regimens to optimize the therapeutic index.

FXR agonists (e.g., INT-747/OCA) demonstrate therapeutic potential in attenuating sepsis-induced cholestasis and intestinal injury. In cecal ligation and puncture -induced septic mice, INT-747 upregulates intestinal FXR/FGF15 signalling, preserving gut barrier integrity and reducing hepatic cholestasis (124). Clinically, OCA is approved for PSC treatment (125), with recent advances utilizing ROS-responsive nanoparticles for targeted OCA delivery to enhance efficacy (126). FXR expression was found to be markedly downregulated in samples of primary CCA (127, 128). OCA counteracts this deficit by restoring BA homeostasis via SHP/LRH-1-dependent pathways, suppressing CCA proliferation and migration (127). Critically, ​​heterogeneous FXR expression across CCA subtypes directs subtype-specific therapeutic strategies.​​ ​​In chemotherapy-resistant iCCA models​​, the ​​combination of FXR agonists (e.g., GW4064, CDCA)​​ with standard chemotherapy (cisplatin/gemcitabine) ​​potentiates anti-proliferative and pro-apoptotic responses​​ (129). ​​Conversely, pCCA and dCCA subtypes frequently exhibit low-to-moderate FXR levels​​ (H-score <120), ​​necessitating the priming use of epigenetic modulators​​ (e.g., DNMT inhibitors) ​​to restore agonist sensitivity​​ (78, 130). ​​Concerning carcinogenic risk, chronic FXR activation displays a complex tissue-selective duality:​​ while systemic FXR loss promotes hepatocarcinogenesis and tumor-specific FXR silencing characterizes the HCC/CCA microenvironment (131), hyperactivation within specific gastrointestinal tissues may drive β-catenin-dependent oncogenesis (132). Therefore, the tissue-selective functional duality of FXR signalling necessitates rigorous long-term safety evaluation in forthcoming preclinical and clinical investigations.

Certain genetic mutations indirectly disrupt BA metabolism. For instance, frequent IDH1/2 mutations in iCCA drive 2-hydroxyglutarate accumulation, which epigenetically impairs cell differentiation and may suppress BA synthesis genes (e.g., CYP7A1) via DNA hypermethylation (133, 134). Ivosidenib (AG-120), a selective IDH1 inhibitor, demonstrated improved progression-free survival in the phase III ClarIDHy trial, becoming the first targeted therapy approved for IDH1-mutant CCA (135). HNF4A, a master regulator of hepatobiliary differentiation, directly modulates bile acid synthesis enzymes and transporters (e.g., BSEP/ABCB11) (136, 137). While HNF4A activation may suppress tumor progression by restoring BA homeostasis, direct targeting agents remain unavailable. Despite preclinical evidence supporting BA metabolic reprogramming as a therapeutic strategy for CCA, BA-targeted agents remain largely experimental with limited clinical translation. This gap necessitates large-scale trials and mechanistic studies to rigorously validate efficacy and safety, accelerating their clinical integration.

## 7 Future perspectives for gut-liver axis targeting in CCA

**Figure 4:**
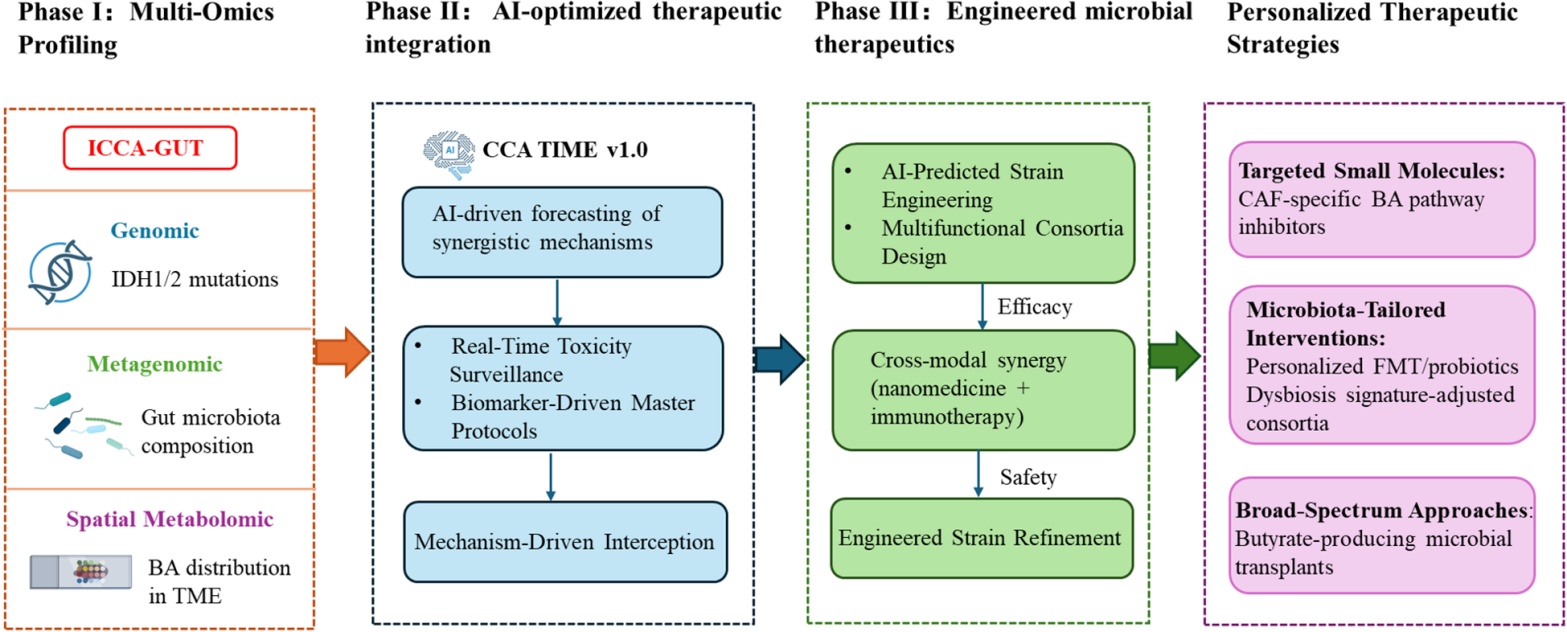
Multistage translational roadmap for gut-liver axis-targeted therapy in cholangiocarcinoma.

The gut-liver axis offers transformative potential for CCA therapy, yet clinical translation requires strategic prioritization to address biological complexity. We propose a phased framework (**Figure 4**) to accelerate clinical impact through defined milestones: Phase I​​ establishes foundational biology through multi-omics validation. Phase II leverages these validated biomarkers to optimize therapeutic strategies using artificial intelligence (AI) -driven modeling of efficacy-toxicity landscapes. Phase III engineers precision interventions targeting CCA heterogeneity—notably cancer-associated fibroblast (CAF)-bile acid signaling inhibition or butyrate-producing microbiome modulation. This continuum from discovery to bioengineered solutions builds an individualized translational pipeline for CCA.

### 7.1 Multi-omics validation (phase I)

The pronounced heterogeneity of CCA necessitates therapeutic targeting based on robust molecular subtyping. Multi-omics approaches—encompassing genomics, metagenomics, and spatial metabolomics—systematically dissect the core mechanisms underlying gut-liver axis dysregulation, including critical microbiota-bile acid-host interactions. These approaches yield quantifiable and targetable biomarkers (e.g., dysbiosis subtypes, cancer-associated fibroblast BA dependency phenotypes) essential for subsequent therapeutic development phases. However, clinically applicable gut-liver axis biomarkers validated through large-cohort studies remain lacking. Phase I prioritizes the validation of foundational biomarkers through global consortia (ICCA-GUT), standardizing profiling to map BA distribution. Leveraging single-cell spatial transcriptomics will reveal cancer-associated fibroblast-specific BA dependencies. Cross-cancer comparative analyses will identify conserved vulnerabilities like pan-hepatobiliary butyrate deficiency. Machine learning frameworks will correlate IDH1/2status with microbiota-metabolome covariation, culminating in a validated dysbiosis subtype classifier (AUC>0.90). This establishes a closed-loop pipeline for refining precision medicine, from multi-omics discovery to mechanism-guided translation.

### 7.2 AI-optimized therapeutic integration (phase II)

Building on validated biomarkers, this phase utilizes AI platforms (CCA-TIME v1.0) to decode the multicellular interactome of tumor microenvironment, identifying therapeutic targets. The platform quantifies multidimensional efficacy-toxicity landscapes, forecasting synergistic interactions (e.g., probiotic-enhanced dendritic cell function augmenting anti-PD-1 responses) (138). Real-time toxicity monitoring flags risks like antibiotic-induced immunotherapy resistance. These insights guide master protocol trials: (1) Arm A targets dysbiosis index ≥5.2 tumors with FMT + anti-PD-1 to correct gut-liver axis dysregulation, and (2) Arm B addresses FXR-methylated subtypes via DNMT inhibitor + OCA combinations that destabilize chemotherapy-resistant microenvironments through cholesterol homeostasis modulation (121). This phase systematically converges microbial engineering, metabolic targeting, and AI-driven design to transition CCA management from empirical to mechanism-guided interception.

### 7.3 Engineered microbial therapeutics (phase III)

Synthetic biology tackles CCA heterogeneity through precision-engineered bacteria with logic-gated (“sense-decide-act”) capabilities, targeting dysregulation via tripartite mechanisms: microbiota modulation, BA metabolic intervention, and antitumor immunity activation (139). Crucially, this phase integrates outputs from prior stage: Phase I-derived single-cell metatranscriptomics enables AI-powered prediction of strain-engineered metabolic perturbations via hepatic flux balance analysis, informing designs like multifunctional consortia (BA regulation), *Lactobacillus reuteri* (IL-22-mediated mucosal repair), and attenuated *Salmonella typhimurium* (hypoxia-targeted gemcitabine delivery). Phase II-validated synergy models (e.g., butyrate-enhanced dendritic cell priming) guide strain-immunotherapy combinations. Convergence with nanomedicine (e.g., liver-tropic nanoparticles co-loaded with FXR agonists/probiotics) or immunotherapies (e.g., anti-PD-1/butyrate) amplifies therapeutic indices (126). Short-term priorities include strain optimization (kill switches, hypoxia-triggered gemcitabine) and ecological integration via butyrate-producing consortia for pan-hepatobiliary malignancies. The pivotal Phase III milestone requires demonstrating ≥30% reduction in CAF, validating engineered microbiota against desmoplastic CCA progression.

## 8 Persistent challenges and knowledge gaps

### 8.1 Mechanistic complexity and model limitations

Therapeutic development for CCA faces significant interspecies translational barriers. Murine models (e.g., KRAS/p53 KO) inadequately recapitulate human cholestatic pathophysiology due to species-specific BA metabolism—notably CYP8B1 deficiency—and the absence of key immune components, including Kupffer cells (140, 141). Further complexity arises from BA-specific receptor (e.g., TGR5) and metabolite (e.g., butyrate) exhibiting context-dependent dual roles (142), necessitating single-cell spatiotemporal resolution to dissect microenvironmental interactions. Biomarker standardization remains elusive, with analytical variability in quantifying >40 carcinogenic BA isoforms and microbial interference with BA gene signatures (e.g., CYP7A1/SLC10A2 ratios) confounding treatment predictions (70). Compounding these challenges, interindividual variability driven by genetic polymorphisms, geographic disparities in microbiota composition (e.g., Bacteroides abundance in Asian versus Western populations) (143, 144), and dietary influences (e.g., high-fat diet-induced TGR5 activation (93)) collectively modulate therapeutic responsiveness, underscoring the need for precision stratification models.

### 8.2 Safety-efficacy dilemmas in gut-liver axis therapeutic

The clinical translation of gut-liver axis modulators faces unresolved safety-efficacy dilemmas. Probiotic-related bacteremia, particularly septicemia caused by *Clostridium butyricum*, is predominantly concentrated in high-risk post-hepatectomy patients (e.g., with neutropenia or bilioenteric anastomoses), contrasting sharply with therapeutic benefits (145). This risk is mechanistically linked to intestinal barrier disruption (e.g., anastomotic leaks) facilitating bacterial translocation. Broad-spectrum antibiotics deplete oncobiotic taxa but concomitantly disrupt chemotherapy efficacy—for instance, by depleting folate-producing *Faecalibacterium*, thereby contributing to 5-FU resistance. This paradox highlights the critical need for optimizing this risk-benefit balance. Synthetic microbial therapies present significant biocontainment challenges. Engineered bacteria (e.g., utilizing the *Escherichia coliNissle* 1917 chassis) demonstrate concerning horizontal gene transfer frequencies (10^-^³–10^-5^ CFU/recipient via plasmids) in murine models, potentially facilitating dissemination of virulence genes (e.g., *ctxfrom Vibrio* cholerae) (146, 147). Furthermore, the limited standardization of FMT protocols and unclear pathogenic thresholds impede clinical reproducibility.

### 8.3 Multi-omics integration hurdles

Current static multi-omics approaches fail to capture the dynamic heterogeneity of CCA, particularly evolving microbiota-immune-metabolite interactions needed for adaptive therapy. While spatial metabolomics and AI enable microenvironment mapping, clinical translation is limited by inconsistent analytical standardization across workflows. The absence of closed-loop systems integrating longitudinal multi-omics data (e.g., IDH-mutation gut-liver signatures) with adaptive algorithms impedes progress from empirical treatment towards AI-driven precision oncology. Addressing this requires collaborative validation of predictive biomarkers through prospective trials and development of real-time monitoring framework.

## 9 Conclusions

The gut-liver axis plays a pivotal role in CCA pathogenesis through dysbiosis-driven microbial metabolite translocation, BA metabolic reprogramming, and immune microenvironment remodeling. Gut microbiota dysbiosis promotes pathogenic endotoxin influx, activating TLR/NF-κB signaling to fuel chronic inflammation and tumorigenesis. Concurrently, BA accumulation drives cholangiocyte proliferation and immunosuppression via FXR/TGR5/S1PR2 pathways, while bidirectional crosstalk between microbial BA metabolism and BA-mediated microbiota modulation perpetuates oncogenic signaling. Therapeutic strategies targeting this axis—antibiotics, probiotics, FMT, and BA receptor modulators—demonstrate potential to disrupt tumor-promoting circuits, particularly when integrated with immunotherapy. Future directions demand multi-omics-guided precision approaches, AI-powered combinatorial regimens, synthetic engineered microbiota to address heterogeneity and receptor duality. Overcoming challenges in mechanistic validation, clinical standardization, and microbiota-host metabolic interplay will be critical for translating gut-liver axis insights into effective CCA therapies.

## Supplementary material

Supplementary Figure 1 Gut microbiota trajectories across advanced cholangiocarcinoma (TNM stage III–IV) and vascular invasion stratification​​.
