# Supplementary figures and images for: Targeting the gut-liver axis in cholangiocarcinoma: mechanisms, therapeutic advances, and future directions
Source: Front Oncol. 2025 Sep 12;15:1646897. doi: 10.3389/fonc.2025.1646897 (PMC12463604; doi:10.3389/fonc.2025.1646897)

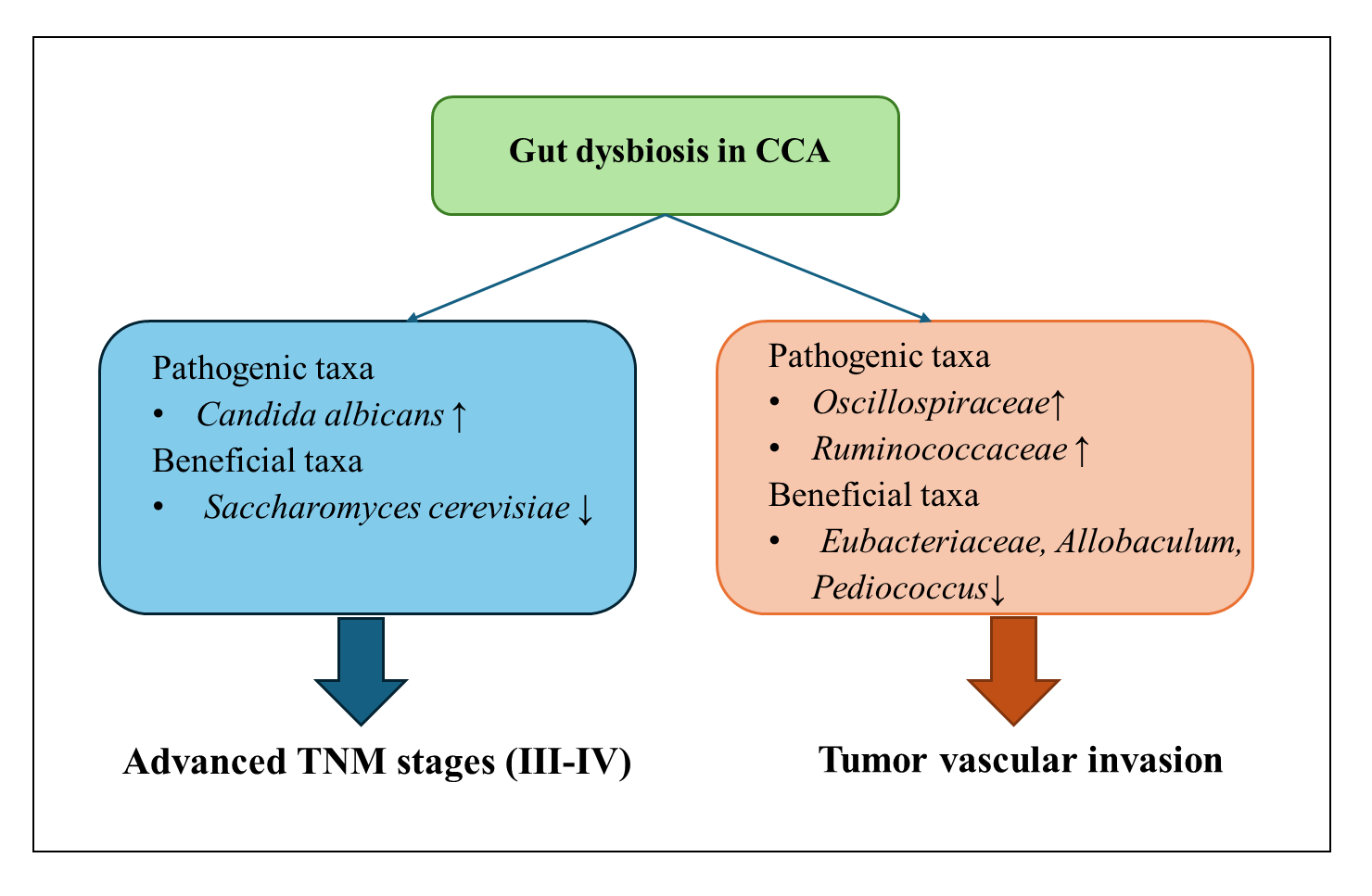

Supplement: Supplementary Figure 1 — Gut microbiota trajectories across advanced cholangiocarcinoma (TNM stage III–IV) and vascular invasion stratification​​. [file Image1.tif]
